# Prevalence and Correlates of Mental Health Outcomes During the SARS-Cov-2 Epidemic in Mexico City and Their Association With Non-adherence to Stay-At-Home Directives, June 2020

**DOI:** 10.3389/ijph.2021.620825

**Published:** 2021-04-21

**Authors:** Mario H. Flores-Torres, Audrey R. Murchland, Priscilla Espinosa-Tamez, Jocelyn Jaen, Marion Brochier, Sergio Bautista-Arredondo, Héctor Lamadrid-Figueroa, Martin Lajous, Karestan Koenen

**Affiliations:** ^1^ Department of Epidemiology, Harvard T. H. Chan School of Public Health, Boston, MA, United States; ^2^ Center for Research on Population Health, National Institute of Public Health, Cuernavaca, Mexico; ^3^ School of Public Health, National Institute of Public Health, Cuernavaca, Mexico; ^4^ Center for Health Systems Research, National Institute of Public Health, Cuernavaca, Mexico; ^5^ Department of Global Health and Population, Harvard T. H. Chan School of Public Health, Boston, MA, United States

**Keywords:** COVID-19, mental health, public healh, anxiety, depression

## Abstract

**Objectives:** To describe the prevalence and correlates of depressive symptoms, generalized anxiety disorder (GAD), and perceived negative mental health impact during the SARS-Cov-2 pandemic in Mexico City and evaluate their association with adherence to stay-at-home directives.

**Methods:** Baseline data from a cohort study of 2,016 Mexico City government employees were analyzed using multivariable logistic regression models.

**Results:** Among participants, 17.2% had clinically significant depressive symptoms, 21.6% had probable GAD, and 15.2% reported that the pandemic has had a major impact on their mental health. Factors including the presence of COVID-19 symptoms, self-isolation, and economic difficulties were associated with poor mental health. The presence of depressive symptoms and general anxiety were associated with non-adherence to public health directives, particularly among those who might have experienced these symptoms for the first time during the pandemic.

**Conclusion:** Our study is one of the first to document the population mental health burden during the SARS-CoV-2 pandemic in Mexico and to provide evidence of the potential role of mental health in the adherence to public health measures.

## Introduction

The SARS-Cov-2 pandemic has killed more than 185,000 people in Mexico as of March 1st, 2021 [1]. Mexico City and the metropolitan area have disproportionately been affected (32% of total cases and 29% of total deaths) [2, 3]. Between March 23rd and March 31st, physical distancing measures (e.g. banning public gatherings and closing schools and non-essential businesses) were introduced in Mexico City, and a national stay-at-home directive until May 30th was issued [4]. On June 1st, a nation-wide step-wise reopening system was introduced [5]. However, Mexico City did not alter mitigation strategies until June 29th when a gradual relaxing of these measures began but a stay-at-home recommendation remained in place.

In addition to its impact on physical health, SARS-Cov-2 may affect mental health through direct (e.g., fear about becoming infected) [6, 7] or indirect (e.g., social isolation because of social distancing measures) stressors [7]. Initial work shows that the pandemic may be exerting an important toll on the prevalence of several mental health conditions including perceived negative impact on mental health (13–29%) [8], generalized anxiety disorder (GAD) (35.1%) [9], depressive symptoms (20.1%) [9], and posttraumatic stress disorder (PTSD) symptoms (7%) [10]. Evidence on the impact of the SARS-Cov-2 pandemic on mental health in Mexico in peer-reviewed literature is limited to a few studies reporting high prevalences of depressive, anxiety [11], and PTSD symptoms [12].

Poor mental health may play a role in shaping the spread of infectious disease through non-compliance with recommended medical treatment [13] and non-adherence to public health measures [14–16]. And while, during epidemics, individuals may be more likely to adhere to public health advice when they are more concerned [17], increasing distress may exacerbate mental health problems, and eventually undermine adherence [18]. To our knowledge, there is no available evidence of the potential impact of mental health on adherence of physical distancing measures in the current pandemic.

Thus, we aimed 1) to provide estimates of the prevalence and correlates of depressive symptoms, GAD, perceived negative mental health impact, and use of mental health services during the SARS-Cov-2 epidemic in a sample of Mexico City’s government workers and 2) to evaluate the relation between mental health conditions and adherence to stay-at-home directives.

## Methods

### Participants

The Study of Urban Health and Social Distancing (or SUSana, in Spanish) is a longitudinal cohort study of Mexico City’s government employees initiated in June 2020 to 1) evaluate health and behaviors during the SARS-Cov-2 epidemic and 2) investigate the impact of these factors on the adherence to mitigation strategies. Between June 4th and July 8th^,^ 2020, Mexico City government employees responded to an online questionnaire (platform developed by Amazon Web Services Mexico-Grupo SIAYEC) that assessed demographic characteristics, pre-existing health conditions, financial difficulties during the pandemic, COVID-19 symptoms, adoption of preventive and social distancing measures, knowledge of the virus, and mental health conditions. Potential participants included all employees with access to an institutional email–those performing any type of desk work in the central government (assistants, administrators, program coordinators, and executive officers). Employees from Mexico City’s Ministry of Health were not included in our sample. Invitations were sent to institutional emails of 14,361 eligible employees; 16.6% (*n* = 2,386) participated in the study. Study participants were younger (mean age, 42.6 vs. 46.9 years) and more likely to identify as women (49.8% vs. 41.2%) than in the eligible source. For the present cross-sectional analysis, we included participants with complete responses to the mental health questionnaire (*n* = 2,139; 89.6%). Among them, a low proportion of missingness (≤2%) was observed for all covariates with no specific pattern (e.g., monotone); missingness did not appear to depend on mental health outcomes (Supplementary Appendix Table S1). Therefore, we conducted a complete case analysis excluding participants with missing covariates resulting in a final analytic sample of 2,016 participants.

### Assessment

#### Mental Health Measures

Depressive symptoms were measured using the Spanish version of the 7-item Center for Epidemiologic Studies Depression (CESD-7) scale [19] querying depressive symptom presence and frequency in the past week (could not get going, trouble keeping mind on tasks, depressed, took extra effort, restless sleep, pleasure, feeling sad) on a four-point scale ranging from 0 (never) to 3 (most of the time). The total score is the sum of the seven items (reverse-coding “pleasure”) ranging from 0 to 21 with higher scores indicating a higher degree of depressive symptomatology. Clinically significant depressive symptoms were operationalized as >8 CESD-7 score based on previous benchmarks used in the Mexican population [19]. This scale has been used in Mexican national surveys [20] and internal consistency was found to be good (Cronbach’s alpha = 0.86) in our study population.

The prevalence of general anxiety symptoms were assessed using the Spanish version of the Generalized Anxiety Disorder-7 (GAD-7) scale [21, 22]. The GAD-7 assesses the frequency of anxiety symptoms (feeling nervous or on edge, not being able to control worrying, excessive worrying, trouble relaxing, restless, irritable, feeling afraid) over the past two weeks on a four-point scale ranging from 0 (never) to 3 (nearly every day). The total score ranges from 0 to 21 with higher scores indicating more severe functional impairments as a result of anxiety. Probable GAD was operationalized using >9 points, based on previously used benchmarks [21]. The internal consistency of this scale in our study population was excellent (Cronbach’s alpha = 0.92).

Perceived negative mental health impact of the epidemic was evaluated based on a question used in the Kaiser Family Foundation Health Tracking Poll [8], “Do you feel that worry or stress, related to the coronavirus has had a negative impact on your mental health?”. Possible responses included, “Yes, a major impact,” “Yes, a minor impact,” and “No.” We also participants asked if they had sought support from available mental health services.

#### Stay-At-Home Directives Adherence

Participants were asked about the number of days they left the household in the previous week. Those who reported going out at least once were further asked about the reasons for leaving the household: groceries, work, medical attention, recreational physical activity, walking their pet, taking care of a relative, social gathering, boredom, not believing in social distancing, and others. Participants were additionally asked to report whether or not they received visits from non-household members in the week previous to the survey. Non-adherence to stay-at-home orders was defined as leaving household for non-essential activities (social gathering, boredom, do not believe in social distancing) or home visits by non-household members.

#### Other Covariates

Measures of sociodemographic factors included age at the time of survey response, sex, education level (less than university, university, and graduate degree), whether participants lived alone or with other household members, monthly household income in Mexican pesos (<$17,500, $17,500-$31,500, >$31,500, and preferred not to respond), and pre-existing physician-diagnosed health conditions (diabetes, hypertension, pulmonary disease, heart disease, depression, and anxiety).

Disease-specific stressors included the presence of at least one COVID-19 symptom in the week previous to the survey (fever, dry cough, shortness of breath, aches and pains, headache, loss of smell or taste, sore throat, and diarrhea or vomiting) and self-isolation due to participant symptomatology or recent contact with a symptomatic individual. Stressors related to mitigation strategies included whether or not participants went out to work in the previous week, presence of financial difficulties in the household during the pandemic (e.g., salary reduction), and inability to take care of themselves or family members (e.g., inability to take care of children at home).

Participants were also asked about the source of information that they consider the most reliable during the pandemic traditional media (television, radio, printed journals), online media (social media and websites), personal sources (physician, friend or family member, religious leader), and official government websites.

### Statistical Analysis

For description of the population, we calculated means and standard deviations for continuous variables, and percentages for categorical variables. Clinically significant depressive symptoms and probable GAD were operationalized based on previous benchmarks used in the Mexican population [19, 21]. Perceived negative mental health impact was dichotomized into no impact/minor impact and major impact. We estimated the overall prevalence of clinically significant depressive symptoms, probable GAD, perceived major mental health impact, and the use of psychological services in the study population and conducted chi-square tests to explore differences across subgroups. Multivariable logistic regression models were used to evaluate the relative importance of different correlates with mental health outcomes (clinically significant depressive symptoms, probable GAD, and perceived major mental health impact).

To estimate the adjusted odds ratio of non-adherence to stay-at-home orders according to the presence of 1) clinically significant depressive symptoms and 2) probable GAD, we used logistic regression models adjusting for theoretically appropriate confounders (Supplementary Appendix Figure S1). Confounders were selected based on subject matter expertise and literature suggesting these measures may be predictors of mental health and non-adherence to stay at home directives [13, 14, 16]. Specifically, our models were adjusted for age, sex, education level, living alone, household income, presence of COVID-19 symptoms in the past week, self-isolation in the past week, going out to work in the past week, financial difficulties in household, not being able to take care of oneself or family members, and history of physician-diagnosed depression or anxiety. Perceived major mental health impact was considered in exploratory analyses.

We repeated analyses among participants with no previous medical diagnosis of depression or anxiety in order to isolate the estimated association among those with recent onset of mental health conditions. Since the pandemic could have a different impact in those with pre-existing mental health disorders [23], compared to those with no disorders, we explored whether the association between mental health conditions and non-adherence to directives varied by history of physician-diagnosed depression or anxiety on the multiplicative and additive scales. We used likelihood ratio tests for multiplicative interactions and the relative excess risk due to interaction (RERI) for additive interactions using Mathur’s et al. R function [24].

All analyses were conducted using R version 3.6.1.

## Results

Characteristics of study participants are presented in Table 1. Sociodemographic characteristics appear heterogeneous in the study population. Among participants, 26.4% reported having at least one symptom of SARS-CoV-2 infection in the previous week (4.2% of participants actually received testing and 0.6% overall received a positive test) and 11.6% of participants self-isolated in this same period. Over half (49.2%) of participants reported financial difficulties in their household since the pandemic started and 19.3% reported not being able to take care of themselves or their families.

**TABLE 1:** Characteristics of participants (*n* = 2,016), Study of Urban Health and Social Distancing, Mexico City, 2020.

Age, mean (SD)	42.6 (12.1)
18–34	600 (29.8)
35–44	547 (27.1)
45–54	483 (24.0)
≥55	386 (19.1)
Sex	
Men	1,013 (50.2)
Women	1,003 (49.8)
Education level	
Less than University	518 (25.7)
University	1,130 (56.1)
Graduate degree	368 (18.3)
Living alone	104 (5.2)
Household income, Mexcian Pesos/month	
<$17,500	763 (37.8)
$17,500–$31,500	565 (28.0)
>$31,500	355 (17.6)
Declined response	333 (16.5)
Preexisting health conditions^a^	467 (23.2)
Previous diagnosis of depression and/or anxiety	189 (9.4)
Had COVID-19 symptoms in the past week	533 (26.4)
Self-isolated in the past week	233 (11.6)
Went out to work in the past week	878 (43.6)
Financial difficulties in household^b^	991 (49.2)
Not able to take care of oneself or family members^c^	390 (19.3)
Most reliable source of information	
Government website	568 (28.2)
Online media (social media and news website)	318 (15.8)
Traditional media (T.V., radio and newspaper)	645 (32.0)
Personal sources (friends and relatives, health care provider or religious leader)	485 (24.1)

Values are *n* (%), unless otherwise indicated.

^a^ Pre-existing health conditions included diabetes, hypertension, and any cardiovascular or pulmonary disease.

^b^ Financial difficulties was defined as having a household member who stopped receiving income in the past two weeks, whose salary was reduced, or who lost their job.

^c^ Not able to take care of themselves or family members was defined as reporting any of the following: water not available in household, soap or hand sanitizer too expensive or not available, facemasks too expensive or not available, cannot take care of children at home, or unavailability of space to isolate those with COVID-19 symptoms.

Mean (SD) CESD-7 and GAD-7 scores were 4.6 (4.5) and 5.9 (5.1), respectively. Among participants, 17.2% had clinically significant depressive symptoms, 21.6% had probable GAD, and 15.2% reported that the pandemic has had a major impact on their mental health. However, among those with clinically significant depressive symptoms, only 31.2% reported seeking any type of psychological care during the epidemic. Comparatively, 25.9% of those with probable GAD and 27.7% of those who reported major mental health impact reported seeking psychological attention.

**FIGURE 1:**
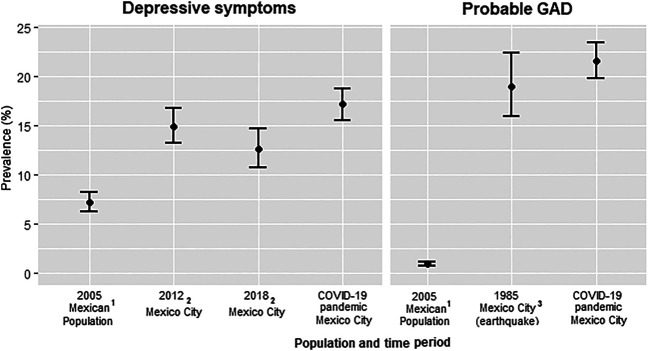
Prevalence of depressive symptoms and probable generalized anxiety disorder (GAD) in the current sample (COVID-19 pandemic) vs. previous population estimates in Mexico (national) and Mexico City, Study of Urban Health and Social Distancing, Mexico City, 2020. References 1 [26]; 2 [25]; 3 [27].

The prevalence of clinically significant depressive symptoms and probable GAD observed in this sample were higher than previous estimates for Mexico City and for the country (Figure 1). Specifically, the prevalence of depressive symptoms observed in our study was higher than previous population estimates in Mexico City in 2012 and 2018 (17.2% vs. 14.9% and 12.6%) [25] as well as nationwide estimates (17.2% vs. 7.2%) [26]. Further, the prevalence of GAD in our sample was much higher than nationwide estimates in 2005 (21.6% vs. 0.9%) [26] and even slightly higher than that reported after the 1985 Mexican earthquake (21.6% vs. 19%) [27].

Prevalence of mental health outcomes differed according to population subgroups (Table 2). The burden of depression and anxiety appeared to be higher in participants with previous diagnosis of depression or anxiety, who self-isolated, had COVID-19 symptoms, and those with difficulties taking care of themselves or their families. In contrast, participants who left their household to work in the previous week had a lower prevalence of mental health outcomes. Poor mental health also appeared to be consistently higher among those who considered online media as the most reliable source of information about the epidemic.

**TABLE 2:** Prevalence of mental health conditions during the SARS-CoV-2 epidemic in the Study of Urban Health and Social Distancing, Mexico City, 2020.

	Depressive symptoms (CESD-7 > 8)	*p*-value	Generalized anxiety (GAD-7 > 9)	*p*-value	Major mental health impact	*p*-value
Study population, % (95% confidence interval)	17.2 (15.5, 18.8)		21.6 (19.8, 23.4)		15.2 (13.7, 16.8)	
Age groups		<0.001		<0.001		<0.001
18–34	27.5		33.2		23.2	
35–44	17.0		23.0		14.1	
45–54	11.2		14.1		12.2	
≥55	8.8		11.1		8.3	
Sex		<0.001		<0.001		<0.001
Men	11.9		16.8		12.4	
Women	22.4		26.5		18.0	
Education level		0.017		0.750		0.230
Less than university	13.1		20.5		16.6	
University	18.3		21.9		15.5	
Graduate degree	19.3		22.3		12.5	
Living alone		0.400		0.038		0.427
No	17.0		22.1		15.4	
Yes	20.2		13.5		12.5	
Household income, Mexcian pesos/month		0.928		0.335		0.003
<$17,500	17.7		23.6		19.0	
$17,500-$31,500	17.2		19.8		13.6	
>$31,500	16.1		20.0		12.7	
Preferred not to respond	17.1		21.9		12.0	
Pre-existing health conditions^a^		0.872		0.073		0.672
No	17.2		20.7		15.0	
Yes	16.9		24.6		15.8	
Previous diagnosis of depression and/or anxiety		<0.001		<0.001		<0.001
No	14.0		18.8		13.1	
Yes	47.6		49.2		36.0	
Had COVID-19 symptoms in the past week		<0.001		<0.001		<0.001
No	13.3		16.5		12.6	
Yes	28.0		36.0		22.5	
Self-isolated in the past week		<0.001		<0.001		<0.001
No	15.0		19.5		14.0	
Yes	33.5		37.8		24.5	
Went out to work in the past week		<0.001		0.012		0.037
No	20.7		23.6		16.7	
Yes	12.5		19.0		13.3	
Financial difficulties in household^b^		0.019		<0.001		0.004
No	15.2		16.8		13.0	
Yes	19.2		26.6		17.6	
Not able to take care of oneself or family members^c^		<0.001		<0.001		<0.001
No	14.1		18.3		13.5	
Yes	29.7		35.4		22.6	
Most reliable source of information		0.033		0.144		0.112
Government website	17.3		20.6		13.2	
Online media	22.0		25.5		18.2	
Traditional media	14.4		19.5		14.1	
Personal sources	17.5		23.1		17.1	

*p*-values were estimated using chi-squared test.

^a^ Pre-existing health conditions included diabetes, hypertension, and any cardiovascular or pulmonary disease.

^b^ Financial difficulties was defined as having a household member who stopped receiving income in the past 2 weeks, whose salary was reduced, or who lost their job.

^c^ Not able to take care of themselves or family members was defined as reporting any of the following: water not available in household, soap or hand sanitizer too expensive or not available, facemasks too expensive or not available, cannot take care of children at home, or unavailability of space to isolate those with COVID-19 symptoms.

Results from multivariable logistic regression analyses were consistent with univariate results for depressive symptoms and probable GAD (Table 3). The strongest associations were observed for those who had a medical diagnosis of depression or anxiety, those with COVID-19 symptoms in the previous week, and those who had difficulties taking care of themselves or their family. Further, compared to those aged 18–34 years, participants in older age groups had significantly lower odds of depressive symptoms, probable GAD, and major mental health impact.

**TABLE 3:** Correlates of mental health conditions during the SARS-CoV-2 epidemic in the Study of Urban Health and Social Distancing, Mexico City, 2020.

	Depressive symptoms (CESD-7 > 8)		Generalized anxiety (GAD-7 > 9)		Major mental health impact	
	Odds ratio (95% confidence interval)	*p*-value	Odds ratio (95% confidence interval)	*p*-value	Odds ratio (95% confidence interval)	*p*-value
Age groups						
18–34	ref		ref		ref	
35–44	0.52 (0.38, 0.71)	<0.001	0.57 (0.43, 0.75)	<0.001	0.54 (0.39, 0.75)	<0.001
45–54	0.39 (0.26, 0.55)	<0.001	0.33 (0.23, 0.45)	<0.001	0.47 (0.33, 0.67)	<0.001
≥55	0.35 (0.22, 0.54)	<0.001	0.28 (0.19, 0.42)	<0.001	0.33 (0.21, 0.52)	<0.001
Sex						
Men	ref		ref		ref	
Women	1.58 (1.21, 2.07)	<0.001	1.37 (1.08, 1.75)	0.009	1.20 (0.92, 1.56)	0.187
Education level						
Less than university	ref		ref		ref	
University	1.48 (1.05, 2.51)	0.027	1.03 (0.76, 1.40)	0.873	0.92 (0.67, 1.28)	0.620
Graduate degree	1.60 (1.03, 2.51)	0.038	1.13 (0.76, 1.69)	0.545	0.76 (0.48, 1.19)	0.229
Living alone						
No	ref		ref		ref	
Yes	1.39 (0.77, 2.42)	0.250	0.66 (0.34, 1.19)	0.189	0.91 (0.46, 1.67)	0.783
Household income						
<$17,500	ref		ref		ref	
$17,500-$31,500	0.93 (0.66, 1.31)	0.683	0.83 (0.61, 1.14)	0.257	0.71 (0.51, 1.00)	0.051
>$31,500	0.91 (0.60, 1.39)	0.671	0.93 (0.63, 1.35)	0.692	0.72 (0.47, 1.09)	0.208
Preferred not to respond	0.95 (0.65, 1.38)	0.780	0.89 (0.63, 1.26)	0.521	0.56 (0.37, 0.84)	0.005
Preexisting health conditions^a^						
No	ref		ref		ref	
Yes	1.16 (0.83, 1.60)	0.372	1.61 (1.21, 2.15)	0.001	1.23 (0.89, 1.69)	0.208
Previous diagnosis of depression and/or anxiety						
No	ref		ref		ref	
Yes	4.31 (3.05, 6.08)	<0.001	3.35 (2.39, 4.71)	<0.001	3.17 (2.23, 4.49)	<0.001
Had symptoms in the past week						
No	ref		ref		ref	
Yes	1.64 (1.22, 2.21)	<0.001	2.03 (1.56, 2.65)	<0.001	1.51 (1.11, 2.03)	0.007
Self-isolated in the past week						
No	ref		ref		ref	
Yes	1.56 (1.07, 2.24)	0.018	1.32 (0.93, 1.85)	0.120	1.25 (0.85, 1.82)	0.258
Went out to work in the past week						
No	ref		ref		ref	
Yes	0.63 (0.48, 0.83)	<0.001	0.91 (0.71, 1.17)	0.474	0.93 (0.71, 1.22)	0.607
Financial difficulties in household^b^						
No	ref		ref		ref	
Yes	1.18 (0.90, 1.54)	0.235	1.55 (1.22, 1.98)	<0.001	1.19 (0.91, 1.55)	0.213
Not able to take care of oneself or family members^c^						
No	ref		ref		ref	
Yes	2.04 (1.52, 2.74)	<0.001	1.71 (1.30, 2.25)	<0.001	1.31 (0.96, 1.77)	0.082
Most reliable source of information						
Government website	ref		ref		ref	
Online media	1.37 (0.93, 2.00)	0.109	1.26 (0.88, 1.79)	0.207	1.44 (0.97, 2.13)	0.072
Traditional media	1.01 (0.72, 1.43)	0.954	1.06 (0.78, 1.46)	0.693	1.15 (0.81, 1.64)	0.435
Personal sources	0.94 (0.66, 1.33)	0.721	1.04 (0.76, 1.43)	0.802	1.29 (0.90, 1.84)	0.164

^a^ Pre-existing health conditions included diabetes, hypertension, and any cardiovascular or pulmonary disease.

^b^ Financial difficulties was defined as having a household member who stop receiving income in the past 2 weeks, whose salary was reduced, or who lost their job.

^c^ Not able to take care of themselves or family members was defined as reporting any of the following: water not available in household, soap or hand sanitizer too expensive or not available, facemasks too expensive or not available, cannot take care of children at home, or unavailability of space to isolate those with COVID-19 symptoms.

Associations between mental health conditions and non-adherence to stay-at home directives (Table 4) revealed that participants with clinically significant depressive symptoms were more likely to leave their household for non-essential reasons or have visitors compared to those with no significant depressive symptoms after adjusting for sociodemographic characteristics, the presence of COVID-19 symptoms and self-isolation, going out to work, financial difficulties, and care difficulties (OR = 2.10 (95% CI: 1.30, 3.35)). Further adjustment for medical history of diagnosed depression or anxiety slightly increased the magnitude of the association (OR = 2.19 (95% CI: 1.33, 3.54)). The association between probable GAD and non-adherence to stay-at-home directives was in the same direction as that of depressive symptoms but did not reach statistical significance. Similar results were observed in exploratory analyses for perceived major mental health impact (Supplementary Appendix Table S2).

**TABLE 4:** Odd ratios and 95% confidence interval for non-adherence to stay-at-home directives according to the presence of mental health conditions, Study of Urban Health and Social Distancing, Mexico City, 2020.

	Cases/Non-cases	Odds ratio (95% Confidence interval)			
		Model 1	Model 2	Model 3	Model 4
Overall, *n* = 2,016					
CESD-7 > 8					
No	71/1,599	Ref	ref	ref	ref
Yes	33/313	2.20 (1.38, 3.46)	2.29 (1.42, 3.62)	2.10 (1.30, 3.35)	2.19 (1.33, 3.54)
GAD-7 > 9					
No	73/1,507	Ref	ref	ref	ref
Yes	31/405	1.53 (0.96, 2.42)	1.57 (0.97, 2.50)	1.57 (0.96, 2.52)	1.58 (0.96, 2.55)

Model 1: age, sex, education level, living alone, household income, and pre-existing health conditions.

Model 2: model 1 + presence of COVID-19 symptoms and self-isolation.

Model 3: model 2 + going out to work, financial difficulties in household, and not being able to take care of oneself or family members.

Model 4: model 3 + physician-diagnosed depression or anxiety.

Analyses restricted to participants with no previous medical diagnosis of depression or anxiety similarly revealed that having clinically significant depressive symptoms without history of previous medical diagnosis of depression was associated with non-adherence to stay-at-home directives. After multivariable adjustment, this association was stronger in magnitude than that observed in the complete sample (OR = 2.54 (95% CI: 1.50, 4.21)). Probable GAD was also significantly associated with non-adherence to stay-at-home directives among those with no previous medical diagnosis of depression or anxiety (OR = 1.85 (95% CI:1.09, 3.06)).

We further explored effect heterogeneity by history of medical diagnosis of depression or anxiety for the association between mental health conditions and non-adherence to stay-at-home directives. The results indicate that the association between mental health conditions and non-adherence to stay-at-home directives was stronger and only significant among those with no prior diagnosis of depression or anxiety (Supplementary Appendix Results).

## Discussion

Our survey of government employees in Mexico City during the SARS-CoV-2 pandemic revealed several important findings. First, we found consistent indicators of poor mental health and suboptimal use of psychological services. Compared to previous literature, the prevalence of poor mental health in our study population was higher than previous population estimates in Mexico City, including in periods following major natural disasters. Second, clinically significant depressive symptoms and probable GAD were particularly elevated among those who had a medical diagnosis of depression or anxiety, those who had COVID-19 symptoms in the previous week, and those who had difficulties taking care of themselves or their family. Finally, we found that elevated depressive or general anxiety symptoms were associated with non-adherence to stay-at-home directives. Most striking, this association was strongest among those with no history of medical diagnosis of depression or anxiety for whom poor mental health might be of more recent onset.

Our study is the first of which we are aware to document an association between poor mental health and adherence to public health directives during COVID-19, particularly among those with recent onset of these conditions. It has been previously proposed that, during epidemics, psychological reactions to stressors and public health directives may play a role in shaping the spread of disease and in worsening the mental health among vulnerable groups [14, 15]. Some studies suggest that mental health conditions affect compliance with medical treatment [13] and adherence to public health measures [16]. In addition, persons with pre-existing mental health disorders may also be more vulnerable to the psychological effects of public health measures such as quarantine and isolation [14, 18]. The relationships between public health measures, mental health, and individual behaviors during epidemics are complex, likely time-varying, and beyond the scope of our data. Our results may suggest that adherence to public health directives might be particularly difficult among individuals with a recent onset of depressive or anxiety symptoms, whereas those with pre-existing depression or anxiety whose symptoms might have increased or recurred during the pandemic may be less likely to go out and receive visits, making access to psychological care more challenging. Due to the cross-sectional nature of our data, however, temporality cannot be established, and future work should aim to elucidate the directionality of these relationships. Regardless, our results suggest public health strategies aimed to control the spread of the pandemic must be coupled with efforts to manage the pandemic’s effect on mental health [6, 16, 28].

To our knowledge, only two other studies have evaluated the impact of the pandemic on mental health in the Mexican population and were conducted primarily among students and employees of private universities [11, 12]. Ramírez et al. [12] reported the presence of psychological distress and post-traumatic stress symptoms in over a quarter of their study sample (22.0% and 27.7%, respectively). Similarly [11], reported a prevalence of depressive and anxiety symptoms of 15.7% and 22.6%, respectively, during April 2020. Using data from a socioeconomically diverse sample, our study adds to these findings, suggesting that depressive symptoms and probable GAD are part of the mental health burden of the pandemic in Mexico.

While differences in measurements across studies may limit comparability of our prevalence estimates, the increased burden of poor population mental health in Mexico during the pandemic compared to previous estimates, including during other major disasters, is consistent with literature examining these trends in other settings [29, 30]. For example, a study in the United Kingdom (United Kingdom) found that mental distress one month after lockdown among individuals aged 16 and older was higher than expected had trajectories from 2014–2019 continued [29]. Similarly, in the United States (US) in April and May 2020, adults were more than three times as likely to present with clinically significant depressive symptoms and general anxiety compared to US adults in 2019 [30].

Likewise, the disproportionate burden of mental health conditions across sociodemographic subgroups in our study population, namely among women and younger age groups, is also consistent with recent findings in other settings [9, 10, 12, 31]. The pandemic has been shown to disproportionately affect women through unequal childcare and domestic responsibilities [32] and increased mental health risk factors such as domestic violence [33]. Similarly, it has been proposed that the economic and social impact of the pandemic disproportionately affects young people [34], an elevated burden of suicidal ideation among US young adults has been recently reported [35].

The association between economic burden and inability to care of self or others with poor mental health is consistent with the literature in other settings [36] and provides a clear target for public health interventions. Similarly, individuals with actual or potential SARS-CoV-2 infection may be at higher risk of poor mental health outcomes and it has been proposed that factors such as increased fear, stigma and isolation may play a role in this relationship [6, 18] which could be another potential area for intervention. Finally, although not statistically significant, our findings further suggest a potential impact of media exposure on mental health, which is likely to increase during lockdown and can magnify the fear and anxiety trigger by the pandemic [6].

Our study has several limitations. First, the low response rate may reflect selection into the study population and might have biased our estimates. However, demographic characteristic of respondents appeared to be fairly similar to those of the source population and a study with similar online recruitment strategies in Mexico had comparable response rates (11–12%) [37]. Second, the cross-sectional nature of our study limits our exploratory analyses to observational associations as temporality cannot be established. A longitudinal assessment of our study population currently underway and is likely to improve opportunities to utilize causal inference. Third, measurement error cannot be ruled out. For instance, our measure of non-adherence to public health directives did not clarify whether or not visits were considered essential or non-essential. Moreover, due to the self-reported nature of this measure, it was not possible to verify whether those reporting adherence instead went out of home for non-essential reasons and/or received visits; it is possible that government employees, in particular, might have underreported non-adherence to public health directives. However, this outcome misclassification is likely nondifferential with respect to measures of mental health and would result in attenuation of the estimates. In addition, the anonymity of our online survey may at least partially decrease this response bias. Our survey was sent by the National Institute of Public Health and, at enrollment, participants were reassured that their employer would not have access to responses. Phone location data were not available in this study. Fourth, our population estimates may not be generalizable to other populations and communities in Mexico. While our population was relatively diverse in terms of age, education level, and socioeconomic background, our study did not include children, older adults, or health care workers whose mental health may be disproportionately affected by the pandemic. Furthermore, study participants were likely to have higher levels of exposure to the virus than other populations since many government employees were considered essential workers. In fact, 43.6% of participant reported going out to work in the week previous to the survey. Additional research is needed to document the effects of these stressors on population mental health in Mexico during the pandemic.

Our observation that increases in depressive symptoms and general anxiety may affect non-adherence to public health directives, particularly among those who might have experienced these symptoms for the first time during the pandemic, has important implications. First, it provides empirical evidence on the potential role of mental health and stress-related behaviors on the adherence to public health measures during epidemics. This is in line with observations during the Ebola outbreak where fear-related behaviors, such as stigmatizing infected survivors and ignoring medical procedures, were reported to disrupt public health efforts [38]. Second, our study evidences the need for public health directives aiming to contain the spread of the SARS-CoV-2 pandemic to address mental health needs to increase adherence. Complimentary psychosocial programs have been implemented with success in previous epidemics and may be appropriate in this setting [39]. As countries gradually reopen, cases are likely to increase [40] possibly requiring reinstatement of more strict public health directives [41]. Thus, if the pandemic is to be controlled, health authorities need to make mental health services available.

Our study is one of the first to document the population mental health burden during the SARS-CoV-2 pandemic in Mexico and our work highlights many potential targets for public health interventions to support mental health. More work is needed to document the continued and potentially changing influence of the pandemic on Mexico’s population mental health; to elucidate the relationship between mental health, history of mental health, and adherence to public health directives; and to identify important areas and opportunities for public health interventions that can improve population health in Mexico in this difficult time.

## Data Availability

The raw data supporting the conclusion of this article will be made available by the authors, without undue reservation.
