# Supplementary material for: Prevalence and Correlates of Mental Health Outcomes During the SARS-Cov-2 Epidemic in Mexico City and Their Association With Non-adherence to Stay-At-Home Directives, June 2020
Source: Int J Public Health. 2021 Apr 21;66:620825. doi: 10.3389/ijph.2021.620825 (PMC9847494; doi:10.3389/ijph.2021.620825)
Supplement: Supplementary file 1 [file DataSheet1.PDF]

SUPPLEMENTARY APPENDIX

Supplementary Appendix Table S1……………………………………………………………….Page 2

Supplementary Appendix Table S2……………………………………………………………….Page 4

Supplementary Appendix Figure S1………………………………………………………………Page 5

RESULTS………………………..……………………………………………………………Page 6

Supplementary Appendix Table S1. Characteristics of participants with and without complete data (n=2,139), Study of Urban Health and Social Distancing, Mexico City, 2020

	**Incomplete data**	**Complete data**	p-value
**n**	123[d]	2,016	
**Age, mean (SD)**	44.8 (12.9)	42.6 (12.1)	0.067
**Women**	55 (53.4)	1003 (49.8)	0.535
**Education Level**			0.059
Less than university	42 (35.6)	518 (25.7)	
University	58 (49.2)	1130 (56.1)	
Graduate degree	18 (15.3)	368 (18.3)	
**Living alone**	8 (8.3)	104 (5.2)	0.261
**Household income, MXP/month**			0.232
<$17,500	45 (38.5)	763 (37.8)	
$17,500-$31,500	26 (22.2)	565 (28.0)	
>$31,500	19 (16.2)	355 (17.6)	
Preferred not to respond	27 (23.1)	333 (16.5)	
**Preexisting health conditions[a]**	47 (38.2)	467 (23.2)	<0.001
**Previous diagnosis of depression and/or anxiety**	12 (9.8)	189 (9.4)	1.000
**Had covid-19 symptoms in the past week**	24 (19.5)	533 (26.4)	0.111
**Self-isolated in the past week**	18 (14.6)	233 (11.6)	0.376
**Went out to work in the past week**	46 (37.4)	878 (43.6)	0.214
**Financial difficulties in household[b]**	62 (57.4)	991 (49.2)	0.116
**Not able to take care of oneself or family members[c]**	27 (22.0)	390 (19.3)	0.554
**Most reliable source of information**			0.458
Government website	19 (23.8)	568 (28.2)	
Online media (social media and news website)	10 (12.5)	318 (15.8)	
Traditional media (T.V., radio and newspaper)	32 (40.0)	645 (32.0)	
Personal sources (friends and relatives, health care provider or religious leader)	19 (23.8)	485 (24.1)	
**Mental health conditions**			
Clinically significant depressive symptoms	19 (15.4)	346 (17.2)	0.713
Probable generalized anxiety disorder	31 (25.2)	436 (21.6)	0.412

Perceived major mental health impact	25 (20.3)	307 (15.2)	0.165

Values are n (%), unless otherwise indicated. p-values were estimated using chi-squared test for categorical values and t-test for continuous variables. [a]Pre-existing health conditions included diabetes, hypertension, and any cardiovascular or pulmonary disease. [b]Financial difficulties was defined as having a household member who stopped receiving income in the past two weeks, whose salary was reduced, or who lost their job. [c]Not able to take care of themselves or family members was defined as reporting any of the following: water not available in household, soap or hand sanitizer too expensive or not available, facemasks too expensive or not available, cannot take care of children at home, or unavailability of space to isolate those with COVID-19 symptoms. [d]Sample size might vary according to availability of data.

Supplementary Appendix Table S2. Odd ratios and 95% confidence interval for non-adherence to stay-at-home directives according to perceived major mental health impact, Study of Urban Health and Social Distancing, Mexico City, 2020

	Cases/ Non-cases	Odds ratio (95% Confidence interval)			
		Model 1	Model 2	Model 3	Model 4
**Overall, n=2,016**					
Perceived major impact					
No	79/1,630	ref	ref	ref	ref
Yes	25/282	1.75 (1.06, 2.82)	1.78 (1.07, 2.88)	1.75 (1.05, 2.85)	1.79 (1.06, 2.94)

Model 1: age, sex, education level, living alone, household income, and pre-existing health conditions

Model 2: model 1 + presence of COVID-19 symptoms and self-isolation

Model 3: model 2 + going out to work, financial difficulties in household, and not being able to take care of oneself or family members

Model 4: model 3 + physician-diagnosed depression or anxiety

Supplementary Appendix Figure S1. Directed acyclic graph describing the association between probable depression/generalized anxiety and non-adherence to stay-at-home directives, Study of Urban Health and Social Distancing, Mexico City, 2020.

A= Probable depression or GAD

Y= Non-adherence to stay at home directives: going out for non-essential activities (social gathering, boredom, do not believe in social distancing) or receiving visits.

L_1= Sociodemographic factors including age, sex, education level, living alone, household income, and pre-existing health conditions.

L_2= Disease related factors including the presence of COVID-19 symptoms and self-isolation.

L_3= Other factors including going out to work, financial difficulties in household, and not being able to take care of oneself or family members.

L_4= physician-diagnosed depression or anxiety

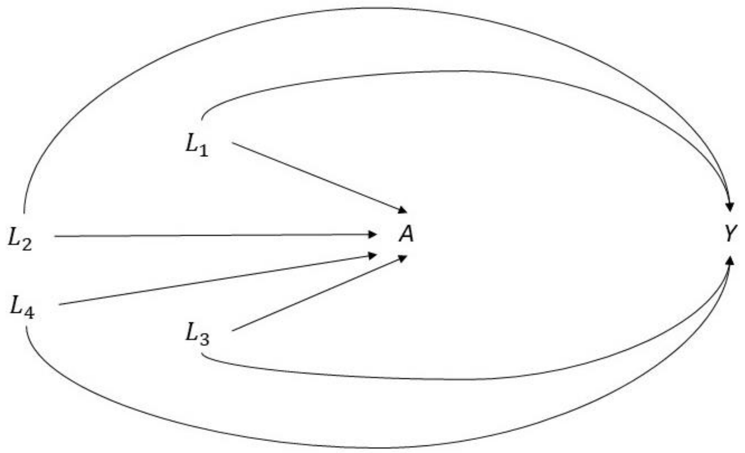

RESULTS

Analyses to assess effect heterogeneity by history of medical diagnosis of depression or anxiety for the association between mental health conditions and non-adherence to stay-at-home orders. For depressive symptoms, stratum-specific odds ratios (95% CI) with a common reference group (no significant depressive symptoms and no history of depression or anxiety) were 1.43 (0.52, 3.29) among those with only previous diagnosis of depression or anxiety, 2.55 (1.51, 4.23) among those with only clinically significant depressive symptoms, and 0.89 (0.27, 2.99) among those with both exposures after multivariate adjustment. In multivariable models, the interaction term (OR=0.35 (0.09, 1.32)) and the RERI (-1.71 (95% CI: -3.80, 0.39)) suggest negative interaction on the multiplicative and additive scales, respectively. For probable GAD, stratum-specific odds ratios (95% CI) with a common reference group (no probable GAD and no history of depression or anxiety) were 1.57 (0.65, 3.36) among those with only previous diagnosis of depression or anxiety, 1.87 (1.11, 3.09) among those with only clinically significant depressive symptoms, and 0.58 (0.16, 2.08) among those with both exposures after multivariate adjustment. In multivariable models, the interaction term (OR=0.31 (95% CI: 0.07, 1.17)) and the RERI (-1.52 (95% CI: -3.35, 0.31)) again suggest negative interaction on the multiplicative and additive scales, respectively.
